# A qualitative study of gutka and paan masala use among Bhutanese and Burmese migrants in Georgia

**DOI:** 10.1371/journal.pone.0237266

**Published:** 2020-08-07

**Authors:** Elizabeth Thai Thanh Do, Milkie Vu

**Affiliations:** 1 Gangarosa Department of Environmental Health, Rollins School of Public Health, Emory University, Atlanta, Georgia, United States of America; 2 Department of Behavioral, Social, and Health Education Sciences, Rollins School of Public Health, Emory University, Atlanta, Georgia, United States of America; University of California San Diego School of Medicine, UNITED STATES

## Abstract

The city of Clarkston (Georgia) is home to many refugees and immigrants, including Bhutanese and Burmese populations. Use of gutka and paan masala is common in these populations. While gutka and paan masala contain toxic ingredients including carcinogens, little research has examined general use, perceptions of risk, cultural norms, and access to these products among Bhutanese and Burmese populations in the southern U.S. This study uses focus groups and key informant interviews to develop an understanding of gutka and paan masala use among Bhutanese and Burmese refugee populations residing in Clarkston, focusing in particular on knowledge and perceptions of harm, patterns of and reasons for use, access to gutka and paan masala, and resources for cessation and prevention of gutka and paan masala use. We conducted 21 focus groups with Bhutanese and Burmese youths and adults and 11 key informant interviews. We analyzed data using MAXQDA and a grounded theory approach. Emerging themes included mixed understandings of ingredients and harms associated with gutka and paan masala use. The continued use of paan masala was perceived to be due to cultural traditions. Youths, particularly Bhutanese, were perceived as a rising group of users of gutka and paan masala. Widespread availability and accessibility in Clarkston made it easy for both adults and youths to acquire and use gutka and paan masala. Few participants knew about prevention efforts or resources in their communities. In conclusion, culturally-relevant awareness and education programs as well as health promotion materials regarding gutka and paan masala are much needed in Bhutanese and Burmese communities. More regulatory actions are needed, such as better warning signs in businesses to inform customers of ingredients in these products and their health risks, age restrictions on gutka and paan masala purchase, and compliance checks.

## Introduction

Tobacco use is the single most preventable cause of death in the world [1]. High levels of tobacco use are observed in disproportionate concentrations among immigrant and refugee populations in the U.S., particularly within South Asian communities [2]. Tobacco products can include smoked products (e.g., cigarettes, cigars, bidis, kreteks, and waterpipes) or smokeless products (i.e., those that could be chewed by the mouth or inhaled by the nose, such as chewing tobacco, moist/dry snuff, dissolvable tobacco, or gutka) [3–5]. The World Health Organization (WHO) estimates there are over 600 million tobacco chewers worldwide with different chewing patterns [6].

Tobacco use is common in South Asia; for example, India is the second largest consumer of tobacco in the world [2]. Tobacco consumption in South Asian culture is characterized by the ubiquity of a variety of both smoked and smokeless indigenous and local tobacco products. In particular, the consumption of smokeless tobacco and areca nut is an integral cultural practice in South Asian countries [7]. These products are becoming more common in the U.S. and can be found in South Asian ethnic enclaves in the U.S. [2]. Particularly, gutka and paan masala are two types of chewing tobacco primarily used among South Asian immigrants in the U.S.

Paan masala (“paan” meaning leaf in many South Asian languages) is the main choice of smokeless tobacco in South Asia, due to its integration into both daily life and culture-based ritualistic customs. Paan is a mixture of various ingredients, including areca nut, slaked lime, spices, seeds and may contain tobacco depending on the user, all wrapped together in a betel leaf or pre-packaged in foil [1, 8, 9]. Areca nut–also known as betel nut–a fruit of the Areca catechu tree (a species of palm), is the main raw ingredient in paan masala, and may be chewed by itself. The key active components of the areca nut are tannins, which are considered both a stimulant and alkaloid [1]. Along with stimulant effects, additional effects such as “improved concentration and relaxation, heightened alertness, diminished hunger, and improved digestion” have also been reported [7]. Areca nut is considered the fourth most commonly used psychoactive substance after caffeine, nicotine, and alcohol [10]. Slaked lime (commonly known as calcium hydroxide) is another raw ingredient commonly found in paan masala and can be obtained from heating seashells, harvested from corals, or quarried from limestone [1]. Ingestion of slaked lime can cause “severe throat pain, a burning sensation in the mouth, abdominal pain, vomiting, bloody stool or vomit, rapidly falling blood pressure and collapse” [11]. Prolonged use of both areca nut and slaked lime in paan masala can cause organ damage [11].

In the past two decades, the tobacco industry in India has aggressively introduced a new product called gutka (also known as gutkha). Gutka, although similar to paan masala, always contains tobacco. Typically, gutka consists of a powdered mixture containing chewing tobacco and other ingredients similar to paan masala (e.g., areca nut, catechu, limes, and spices) packed in thin foiled packets or tins [7, 9, 12, 13]. Gutka has become widely popular in South Asia for its ease of use, low cost, convenient packaging, long shelf life, lack of social stigma, and youth appeal [9]. Many South Asians also believe that gutka is harmless and only acts as a “mouth freshener” [9]. Gutka use typically begins at a young age and is attractive to younger users due to the large amount of sweeteners that are added to conceal the bitterness of the product. Gutka marketing to children is further exacerbated, as children are not socially permitted to smoke cigarettes. Research in India suggested that one out of three children or teenagers regularly or occasionally chewed gutka [7]. As a result, gutka became the only open outlet for many children to engage in tobacco use.

Regular use of gutka and paan masala can lead to oral cancer and other precancerous conditions [14]. According to Changrani & Gany, of the 390,000 oral and oropharyngeal cancers estimated to occur annually in the world, 58% occur in South and Southeast Asia due to the high proportion of South Asians using chewing tobacco [7, 15]. Similar to other chewing tobacco products, gutka and paan masala were shown to have carcinogenic properties and are highly addictive [16]. The WHO classifies smokeless tobacco as carcinogenic to humans [1].

Use of gutka and paan masala is reported among South Asian immigrants in the United Kingdom, South and East Africa, and in parts of Europe and Australia [9]. Chewing tobacco practices have been observed to carry over to second-generation immigrants. In the United Kingdom, occasional use of paan masala among school-aged children also shifts to more regular use of gutka and paan masala upon high school graduation [7].

Research on gutka and paan masala in the U.S. has been very limited, especially among South Asian immigrants. Most research on gutka and paan masala use has focused on Indian and Bangladeshi populations in New York, New Jersey and California [14, 17–19]. For example, Changrani and colleagues administered a survey to understand gutka and paan masala use and beliefs among 183 Bangladeshi and Indian-Gujarati immigrants in metropolitan New York [14]. The study found that while the use of gutka and paan masala migrated with both populations, their perceptions and use of these products significantly differed. Gutka was more popular among Indian-Gujarati immigrants while paan masala was more popular among Bangladeshi immigrants. A second study focusing on 39 South Asian immigrants in New York City explored perceptions of gutka and use patterns using focus groups. Study participants stated that gutka and paan masala were easily accessible in neighborhood stores and noted initiation was influenced by social networks, perceived benefits, and curiosity [17]. The majority of participants initiated the use of gutka and paan masala when they were young (14–17 years old); some other initiated use as young adults (22–26 years old) or later in life (40–45 years old) [17].

Another study with 78 South Asians in Central New Jersey found that paan masala use was more common among elders; the product was used commonly at social gatherings or after meals [18]. By comparison, a study with 2140 participants in California noted that Indian immigrants had lower past-30-day use of conventional tobacco products (4.2%) compared to the general California adult population (14.6%). Conversely, Indian immigrants had higher ever use of traditional tobacco products (64.9%) and a majority (96.6%) had already used these products prior to immigrating to the U.S. [19].

To date, there has been little research done on Burmese and Bhutanese populations in the U.S., who are key populations using gutka and paan masala as evidenced by the use of these products in their home countries [1]. Bhutanese populations began arriving in the U.S. in 2008 and represented 17% of total refugees resettling in the U.S. in 2010. By 2011, that number had increased to 26% of the total number of refugee arrivals [20]. Additionally, Burmese made up the largest refugee group resettled in the U.S. from 2002 to 2011, amounting to 17% of the total 515,350 refugees [20]. The Pew Research Center estimated that from 2008 to 2017, around 67,100 refugees arrived annually in the U.S., with many from Iraq and Burma/Myanmar [21]. Burmese became the second largest refugee population from 2009 to 2011, increasing to represent over 30% of the total number of refugee arrivals in the U.S. [22]. Together, Bhutanese and Burmese refugees became two of the largest refugee groups arriving in the U.S. beginning in 2011.

Many of these refugees face significant challenges as they transition into a new life in the U.S., including poor English language skills and little education or work experience. A study showed that Burmese had the highest high school dropout rate (44%) among all major Asian ethnic groups and 33% of Burmese were living below the poverty line [23]. Religious beliefs, practices, and institutions play a large role in adaptions, community building, and cultural navigations for refugees [22].

The largest Burmese and Bhutanese populations in the U.S. are located in the South, followed by those in the West, Northeast and Midwest [20]. Based on 2014 data, many Bhutanese refugees were resettled in Pennsylvania, Texas, New York and Ohio and a majority of Burmese refugees were resettled in Texas, New York, North Carolina, Indiana [20]. Additionally, the state of Georgia has the fifth highest number of Bhutanese refugees [22]. The 2010 census data showed that Bhutanese and Burmese populations in Georgia were relatively young, with a median age of 26.3 for Bhutanese and 23.4 for Burmese [24]. Around 26% and 7% of Bhutanese and Burmese households in Georgia, respectively, had three or more generations [24].

Within the state of Georgia, the city of Clarkston, known for its diversity, has been accepting displaced persons from many backgrounds (including Bhutanese and Burmese populations) since the 1990s through the U.S. refugee asylum program [25]. Clarkston had a total area of 1.4 miles and an estimated population of over 7,500 based on the 2010 census [26]. Bhutanese and Burmese residents represented over 50% of the Asian population in Clarkston [24]. It is likely that Bhutanese and Burmese populations bring culturally-specific behaviors with them, including gutka and paan masala use; however, there is a dearth of research on this issue.

Because gutka and paan masala are still emerging products in the tobacco market in the U.S. and primarily sold in ethnic enclaves, limited law enforcement and regulation exists regarding these products. In general, current U.S. regulations are sparse at best with respect to gutka and paan masala. Areca nut is placed on a list of herbs that are unacceptable as a non-medicinal ingredient in oral use products [1]. The U.S. Food and Drug Administration currently maintains an import alert and the U.S. government keeps a ban on interstate sales of areca nut [1]. However, the U.S. Bureau of Alcohol, Tobacco and Firearms does permit the legal import of gutka [1]. The manner in which these regulations are enforced by the U.S. government and FDA is unclear. A study conducted in King County, Washington found limited regulations and policies surrounding betel nut use and little to none betel nut sales policy [27].

One U.S. territory has adopted measures that could protect minors from these products. The Commonwealth of the Northern Marina Islands, consisting of 15 islands in the northwestern Pacific Islands, passed the Betel Nut Control Act in 2016 to protect youth from the danger of oral cancer. Under this law, retailers were required to obtain a license to import or sell betel nut within their establishment. This law imposes fines and penalties to any person or business that sells to a minor (under the age of 18). Furthermore, the law prohibits betel nut that are sold within these establishments to be displayed openly to the public [20].

To compare with other countries, the sale of areca nut has been banned due to the link between arecoline and mutagenic effects in Canada [6]. In Australia, the active ingredient in betel nut is considered a Schedule IV poison and is illegal to possess or sell without proper authority [28]. In the European Union, however, there are no specific laws that regulate or ban the sale of areca nut products even when mixed with smokeless tobacco [1].

Youths are exposed to gutka and paan masala within their households if their parents/guardians use them [29]. Within the community, the lack of regulations and thereby lack of law enforcement on gutka and paan masala can further influence use of these products among youth populations. Local business owners will not encounter any risk in providing access to gutka and paan masala without age verification or other restrictions. Since these products are relatively inexpensive, business owners are more relaxed as to who can access these substances. In contrast, tobacco sales laws have been shown to increase compliance among retailers and reduce illegal sales to minors (e.g., through compliance checks in the community) [30, 31]. Furthermore, reducing sales to minor has also been shown to limit accessibility of tobacco products to minors [31].

Product placement of gutka and paan masala plays an integral role in how it is marketed to the community. In some instances, the products are laid out on countertops and are easily seen, thus attracting more customers and catching the attention of youth. In other instances, these products lack proper health risk signage or are advertised “in-language” (i.e., in a non-English language), which contributes to law enforcement missing the products in question completely when they carry out compliance checks on local businesses [32].

To sum up, there has been little research on the extent of use and access to gutka and paan masala among South Asians in the U.S., especially among Bhutanese and Burmese populations. Such information is key for local communities, schools, and law enforcement to create local regulation and awareness or education programs targeting these communities. This study uses focus groups and key informant interviews to develop an understanding of gutka and paan masala use among Bhutanese and Burmese refugee populations residing in Clarkston, GA. Specifically, this study explores knowledge and perceptions of harm, patterns of and reasons for use, access to gutka and paan masala, and resources for cessation and prevention of gutka and paan masala use among Bhutanese and Burmese refugee populations.

## Materials and methods

The interview and focus group data for this study were originally collected as a part of a community program related to substance use prevention among youths from immigrant or refugee backgrounds. The data collected were not identifiable. The Institutional Review Board (IRB) at Emory University determined that this project did not require IRB review.

The focus groups were conducted at the Center for Pan-Asian Community Services (CPACS), a non-profit organization located in Doraville, GA, and assisted by the Georgia Team Empowerment (GATE) coalition, a substance abuse prevention coalition within CPACS that works with Latino and Asian Pacific Islander (API) communities throughout the greater Atlanta, GA area including the cities of Chamblee, Clarkston, Doraville, and Sandy Springs. Participants were selected at two after-school sites in Clarkston, targeting both youths (aged 9–17) and adults (aged 18+) from Bhutan and Burma. Youths were specifically defined in this study as individuals who are younger than 18 years old. Age 18 was chosen as the upper age limit because that age was the legal age for tobacco use at the time of this study [33]. The two after-school sites were managed by youth site managers who assisted in selecting students and adults to participate in the focus groups and interviews. Because CPACS served the API community, many Bhutanese and Burmese clients often visited the site for various social services. Staff members who were trusted among this population assisted in recruiting participants for focus groups and interviews.

Focus group and interview questions were piloted with community members from late December 2017 to January 2018 (i.e., prior to the start of the study) to evaluate whether the questions were sufficiently culturally sensitive and appropriately understood. After interview questions were piloted and refined, focus groups and interviews were held in January 2018 and continued through April 2018. A total of 122 participants were recruited for the focus groups. Twenty-one focus groups (stratified by ethnicity and age group) were conducted, each with at least four to eight participants, lasting from 15 to 60 minutes. The aim of the focus groups was to explore community members’ knowledge and perceptions of harm of gutka and paan masala, patterns of and reasons for use, availability of and access to gutka and paan masala, and resources for prevention and cessation. The first author (ED) served as the only interviewer; she had previous training in qualitative interviews through coursework in her Master’s in Public Health degree. CPACS interpreters who were fluent in English as well as the target language(s) were present during focus groups to assist with translation. The interpreters had been trained in qualitative interviewing and had experiences conducting interviews and focus groups in previous programs and evaluations. During focus groups, CPACS interpreters translated the questions from the interviewer to the target language(s) for focus group participants. Then, interpreters summarized the responses from focus group participants and translated them to English for the interviewer. S1 Appendix lists all the focus group questions used.

Eleven key informant interviews were conducted in from February 2018 to April 2018. The first author (ED) served as the only interviewer. Interviews were held with several community members and leaders, local store and business owners, and local law enforcement officers. Interviews lasted between one-half and one hour each and were conducted in-person. A total of 7 interviews were conducted in English and 4 interviews were conducted in the target language(s). For interviews conducted in the target language(s), CPACS interpreters were present and facilitated translation in a similar manner to the focus groups. Interview questions explored knowledge of risk for each ingredient that is included in gutka and paan masala, personal use, and knowledge of any efforts or policies in the community directed at gutka and paan masala. S2 Appendix lists all the interview questions.

A $10 gift card was given to each focus group and interview participant in exchange for their participation. All focus group and key informant interviews were recorded and transcribed by the first author (ED). Data analysis was conducted using a grounded theory approach to inductively identify analytical categories emerging from the data [34, 35]. The first author (ED) read through the interview transcripts as a whole, indexed data, and identified emerging concepts. From the list of emerging concepts, a codebook was created. The first author (ED) then applied this coding scheme to all transcripts and coded the data using MAXQDA. Codes were then grouped into higher order conceptual themes. The second author (MV) independently reviewed a subsample of 20% of the focus group and interview transcripts to ensure that identified codes and themes were exhaustive.

## Results

A total of 21 focus groups and 11 interviews were conducted. Focus groups were stratified by ethnicity (i.e., Bhutanese or Burmese) and age group (i.e., below 18 years old or 18 years old and above). We conducted 6 focus groups with Bhutanese adults, 4 focus groups with Burmese adults, 1 focus group with both Bhutanese and Burmese adults, 4 focus groups with Bhutanese youths, 5 focus groups with Burmese youths, and 1 focus group with both Bhutanese and Burmese youths. We note that a majority of Bhutan refugees are descendants of Nepalese migrants (referred to as Lhotshampas). Many Lhotshampas fled Bhutan after a government crackdown and settled in Nepalese refugee camps; thus, some Bhutanese individuals may refer to their community as Nepali [36].

### Knowledge and perceptions of harm of gutka and paan masala

Across all ages, the majority of focus group participants indicated that gutka and paan masala products were known in the community. The majority of Bhutanese focus group participants stated they knew about ingredients in both gutka and paan masala. While most Burmese focus group participants stated they did not know about any of the ingredients in gutka, the majority stated they knew about the ingredients in paan masala. When asked about slaked lime and betel nut (i.e., two main components of both gutka and paan masala), the majority of Burmese and Bhutanese focus group participants reported knowing what betel nut and slaked lime were. The majority of key informant interviewees had heard of gutka and paan masala; however, only one interviewee was able to fully explain what slaked lime was, how it was made, and its relevant health effects. Only one interviewee was able to fully explain what betel nut was.

In terms of understanding of health risks, a few Burmese and Bhutanese focus group participants did not know of any health risks associated with gutka or paan masala. Three out of eleven key informant interviewees did not know of health risks associated with gutka. Five out of eleven interviewees either did not know the health risks associated with paan masala or did not perceive paan masala to be harmful.

In terms of perceptions of harm, in almost every Bhutanese and Burmese focus group, participants mentioned some sort of health risks associated with gutka and paan masala use. Most participants reported that gutka and paan masala caused “mouth cancer,” “cancer,” “upset stomach,” “tearing in mouth lining,” and that these products “messes up the teeth.” Focus group participants stated their beliefs that gutka was generally more harmful than paan masala because gutka was marketed mainly as a form of chewing tobacco. They also believed that gutka was more addictive. For example, an adult participant in the Bhutanese focus group stated: “I think [gutka] is kind of like a tobacco product. It's white and it has white stuff… [Paan masala] is the same thing as gutka, It's a little less harmful than the white one. That's all I know and it's a lot more sweeter.” Some adult Burmese focus group participants also stated since gutka was “soaked in alcohol,” it was more dangerous and addictive. One adult Burmese focus group participant suggested that gutka use could cause erectile dysfunction among male users and cause complications for couples who wanted to get pregnant.

Several participants did not believe paan masala to be harmful. For example, an adult Bhutanese focus group participant stated: “I think [paan masala use is] more than gutka since it’s not really that harmful. I have seen little kids eating [paan masala] so I think it’s really common.” Participants described that most often, paan masala ingredients were mixed with honey or coconut to make it sweeter, thereby attracting more consumers, particularly youth. They explained how paan masala was mostly used as a “mouth freshener” after meals and used as medicine to treat coughs and colds. Participants believed that by turning the paan leaf into a liquid, it can treat the common cold and warm the body once applied to the targeted area. They also thought that chewing paan masala or betel nut alone was believed to “strengthen teeth” and that paan masala was less harmful than gutka. One key informant interviewee stated he did not believe there was any harm associated with gutka or paan masala.

To further assess participants’ understanding the three main ingredients in gutka and paan masala, betel nut, slaked lime, and tobacco, focus group participants were asked if they knew anything about the three ingredients. A few Burmese focus group participants stated they believed that betel nut was “good for the teeth.” Participants from one Bhutanese focus group stated that use of betel nut was widely accepted in their culture and religion. Most participants stated that betel nut came from a tree and could make you dizzy if it was not properly prepared. However, participants reported higher perceptions of harm for slaked lime. For example, an adult Bhutanese participant in a focus group said: “[I]f you use a little bit more it takes [away the] outer skin in your mouth. When you add water, it reacts. They believe [chemicals are added] to the stone.” Another youth Bhutanese participant in a focus group stated: “I heard [slaked lime is] really harmful for your mouth. It makes your tongue thick.”

When asked about their knowledge of tobacco, most of the focus group participants thought of tobacco as a component of cigarettes. Other participants had little knowledge about tobacco in comparison to their knowledge of betel nut. Despite this, participants were able to list health effects associated with tobacco use (i.e, cancer, heart, and lung problems). Many youth participants understood the risks of tobacco use and discussed that tobacco use was harmful.

Overall, adult Bhutanese focus group participants displayed a higher level of knowledge and perception of harms of gutka and paan masala than adult Burmese participants. Adult Bhutanese focus group participants were able to describe the ingredients of both gutka and paan masala in more details. Adult Burmese participants were generally more aware of the ingredients and health effects of paan masala but were not as knowledgeable about gutka. Similar results were seen in the youth focus groups. Youth Burmese participants were more familiar about paan masala than gutka in comparison to youth Bhutanese participants who were familiar with both gutka and paan masala. Youth participants in both Bhutanese and Burmese focus groups were in consensus about the harms of using gutka and paan masala and believed the use of these products can adversely affect one’s health and teeth. A handful of both adult and youth participants believed that paan masala was less harmful than gutka due to its sweetness and how ingrained it has been in their culture and religion. In addition, they also believed that the paan leaf itself was medicinal and safe for children to consume.

While two key informant interviewees understood the health risks of gutka and paan masala, they were not fully aware of the ingredients in both products. One interviewee was more knowledgeable about the history of gutka and paan masala due to his past conversations with business owners and community members. Other interviewees were generally aware of the health risks of gutka and paan masala and their ingredients.

### Patterns of and reasons for use

Focus group participants indicated that adults were the main group to use gutka and/or paan masala and attributed continued use to either addiction or out of habit. Most adults mentioned using gutka and/or paan masala as something to chew on after a meal. For example, an adult Burmese focus group participant said: “Some people they are just addicted… when we asked why do you use [gutka], they say I feel bored so I use this to feel better. They feel incomplete after having their lunch or meal, they feel something is missing…” Adult Burmese focus group participants also stated that gutka and paan masala use varies depending on ethnic groups. An adult Burmese focus group participant mentioned: “For the Chin community they eat more gutka, and the Karen community eat more paan masala.”

Culture and tradition of paan masala use was mentioned ten times throughout all of the focus groups and was mentioned once for gutka use. One Bhutanese focus group participant attributed paan masala use to the country’s history: “In the country, our ruler used to eat human being, this paan is like eating human being. Somebody told them this paan it's like human being, leaf is their tongue and nut is their heart and lime is their brain.” One key informant interviewee stated: “Because growing up and our culture, in our tradition, whenever we have visitors or guests at home, [paan masala] is something we offer as a refreshment to your visitor or guest. That's how the tradition started. Just like people give you juice or apple in this country. It's more like a refreshment.” Another key informant interviewee stated: “In our culture and back home in our village, everybody eats [paan masala or gutka]. So then it's more like a friendly gesture, that you offer this to a friend. It's a very kind thing to do back home in the village.” In addition, slaked lime and betel nut were mentioned once each as a cultural norm. An adult Burmese focus group participant stated: “It is our tradition that we make [slaked lime] on our own. In our tradition back home, our ancestors live to a hundred years old because they make their own lime.” Another adult Bhutanese focus group participant stated: “[Betel nut grows in a] tree, it grows in a tree, as for our culture and religion it is accepted everywhere even in religious rituals. So even if you do religious ceremonies or rituals you need [betel nut].”

Participants described that while adults remained the main group of users for both products, youths represent a new rising group of users in Burmese and Bhutanese communities. In particular, Bhutanese youths were perceived to use either product more regularly than Burmese youths and are able to more easily access these products within the community. An adult Bhutanese focus group participant reported; “[I]f they find the money, students who are going to school use [these products] a lot. Usually older folks they don't know this thing and don't pay attention to this. It's a younger generation problem.” The sweetness of the ingredients in paan masala were also perceived to attract youth users. A youth Bhutanese focus group participant said: “[Paan masala is] really tasty. When you first eat it, it's because of the sweetness. It's like a habit. Once you start eating it you can't really quit it whether you want to, it's because of the taste because it's kind of good.” A key informant interviewee also mentioned: “They have one certainly for the kids…instead of tobacco, they use the same leaf and use the paste… they put coconut and jelly beans for kids.” In addition, a key informant interviewee discussed how youths who recently migrated to the U.S. with their families may be at higher risk for using due to a lack of understanding of local rules and regulations: “[I]t’s very likely being used by minors. And unfortunately, this is same culture misunderstanding most of us immigrants have. Because where we grow up there are no age limits, there is no legislation that controls this stuff.”

Most focus group participants stated that gutka and paan masala use was a large problem in their community because youths were more enticed. People also spit out paan masala or gutka in public places, causing red stains on property and littering from used gutka packets. One key informant interviewee explained his concerns of continuous paan masala and gutka use: “From what I see you know wrappers, papers, plastic wrappers thrown everywhere you can see that people are using, [paan masala and gutka] are being widely used and I am especially concerned.”

Questions about personal use were only asked in key informant interviews and not in focus groups. When asked about personal use, four out of eleven key informant interviewees stated they use either gutka or paan masala. Of the four interviewees that use either product, only one interviewee used gutka. All four users stated they used either product every day, and used them when they were alone, after meals, or if they had nothing to do. Three out of four users indicated that they could stop at any time if they wanted to. The rest of the interviewees stated they were “never exposed to it,” did not like the smell, or did not want to damage their teeth.

Adult participants in both Bhutanese and Burmese focus groups believed that the use of gutka or paan masala was out of habit or due to traditions. Culture and traditions play a large role in the continued use of these products as they become more normalized as “mouth fresheners” or offered to guests as refreshments. Adult Burmese focus group participants indicated that use differed by ethnic subgroups as seen between the Chin and Karen communities and that paan masala was generally more popular than gutka. Adult Bhutanese focus group participants believed that youths were more likely to use gutka or paan masala. By comparison, adult Burmese focus group participants believed adults were more likely to use gutka or paan masala. However, adult Burmese focus group participants also understood that youths overall were steadily becoming more likely to use gutka or paan masala due to peer pressure. In particular, the sweetness of paan masala attracted younger consumers.

Youth Bhutanese and Burmese focus group participants believed gutka use was higher among adults, especially among adult males. In addition, youth Bhutanese focus group participants believed gutka was more common in the Nepali community. Some youth participants in both Bhutanese and Burmese focus groups noted that they did not generally regard youths as users of gutka or paan masala. These participants also noted that of the youth users they knew, their use was limited to paan masala. Youth Bhutanese focus group participants were more knowledgeable than their Burmese counterparts and understood the prevalence and reason for use which was mostly attributable to habit and the addictive quality of the products.

Interviews offered more insights into the patterns and reasons of use. Similar to the focus group participants, interviewees discussed the roles of habits, culture, and addictiveness. Further, interviewees stated that the continued use of gutka or paan masala was due to the wide availability of these products in the community. A majority of the interviewees also noted that gutka use was primarily due to the product’s tobacco content. In contrast, paan masala was used more for its believed medicinal properties, sweetness, and its tie to religious practices. Interviewees who were users of either gutka or paan masala attributed their use to peer pressure or out of boredom. Only one user indicated that their use of paan masala was a way to transition out of using conventional tobacco products.

### Access to gutka and paan masala

Most of Bhutanese and Burmese focus group participants mentioned that gutka and paan masala could be bought in Nepali, Burmese, and Indian grocery stores in Clarkston. Most Bhutanese and Burmese focus group participants said that gutka and paan masala were generally “kept in the open” and that it was easy to access these products. Additionally, a few Bhutanese and Burmese focus group participants also mentioned being able to access gutka and paan masala in apartments within Clarkston due to residents making and selling these products in their home. For example, if youth were not able to purchase these products at the stores, there were sellers within apartment complexes who would easily sell them to youth, thus propagating exposure and availability. An adult Burmese focus group participant stated: “In the store if [retailers] don't let the kids buy [gutka or paan masala], but at house if they sell, they let the kid buy it.”

When asked how old individuals needed to be to purchase gutka and/or paan masala, most Bhutanese and Burmese focus group participants indicated that individuals needed to be “18 and older.” However, a few participants stated various other ages, such as “11 to 13 years old,” “15 to 17 years old,” “20 years old,” and “no age restrictions.” In addition, when asked how old individuals needed to be to purchase tobacco, various ages were also stated, with “18 years old” being the most frequently mentioned and “over 21” the second most frequently mentioned. Lastly, the majority of focus group participants said that IDs were not checked when asked if participants ever experienced or witness others getting their ID checked when purchasing gutka and/or paan masala.

Overall, the majority of the adult Bhutanese and Burmese focus group participants knew where to access gutka or paan masala. Adult Bhutanese focus group participants were more knowledgeable about the placement of these products in stores than adult Burmese participants. In addition, adult Bhutanese focus group participants viewed 18 as the legal age to purchase gutka or paan masala. In contrast, adult Burmese focus group participants were unsure of the age to purchase gutka or paan masala. Most of the adult focus group participants stated if youth were unable to buy in the store, they would able to access these products through older friends or could buy elsewhere.

Youth participants in both Bhutanese and Burmese focus groups knew where gutka or paan masala was available in the community and had seen them in stores. Most youth Bhutanese and Burmese focus group participants believed 18 was the age to be able to purchase gutka or paan masala. The majority of youth Bhutanese and Burmese focus group participants also believed that youth access to gutka or paan masala was not easy and that access was easier for adults. Two youth Bhutanese focus group participants believed that youth access to these products was easier either back in their home country or if youths paid off someone to purchase these products for them. Interviewees also gave similar responses as focus group participants. Most interviewees believed it was easy for youths to access gutka or paan masala and either did not know the age to purchase these products or stated there was no age limit. Only two interviewees stated that 18 was the legal age to purchase gutka or paan masala and that youth access was not easy.

### Resources for cessation and prevention

Most participants stated that there is a lack of prevention efforts regarding gutka and paan masala in their communities. When asked if participants knew of any prevention efforts, services and available information available in their communities, every focus group participant indicated not being aware of any or that none were available. While CPACS had released a “No Gutka” public service announcement (PSA) and created a set of campaign posters for gutka and paan masala use in the Bhutanese and Burmese communities, these measures went unnoticed. Focus group participants stated that having a trusted community leader or having someone from a trusted organization come to their community and hold a workshop or in-person training would have a larger impact on increasing the level of awareness in the community. For example, an adult Bhutanese focus group participant stated: “The best way is to stop all [production]. Nepali community has been using so long time, so stop it… Nepali community it is very harmful to them. They want awareness programs… the best way to reach out is weekends and all parents together and let's talk about the harm of all those things. Awareness is important…”

Key informant interviewees reported that it would be difficult to pass or enforce any type of policy or ordinance for gutka and paan masala because these products were so new in the community and the U.S. The widespread availability of the products drove the continued use among Bhutanese and Burmese populations. In addition, other ethnic groups within Clarkston, such as East Africans, were perceived to use the products as well. Key informant interviewees also reported that even if the products were banned, individuals would still able to make and use within their homes. A key informant interviewee stated: “I’m not a fan of prohibiting something because it doesn’t stop the person accessing it. I believe on educating people would be the best thing to do rather than just prohibiting something and trying to control it that way. I think the best way would be educating and partnering with the main community leaders and… church leaders or temple leaders… this is not gonna be easily just legislated… because it’s not a product that is made here in the US… they gonna make it in their own homes, this is gonna be very hard to regulate. I think the best would be definitely focus on educating the people about the ill effects of this specific products.*”*

One key informant interviewee who sits on the city council in Clarkston suggested that it may be better to first educate the entire community on the health risks of using such products and then use this knowledge as a “springboard” to change policy. However, it was emphasized that there should be policies available to control the sale and usage of gutka and paan masala, particularly among youth. In addition, gutka and paan masala could be difficult to generally enforce because they do not technically contain illegal substances. For example, this interviewee stated: “I think the best way would be educating and partnering with the main community leaders and like I said earlier church leaders or temple leaders. So, this is not gonna be easily just legislated and get down with it because it’s not a product that is made produced here in the US or some of it, they gonna make it in their own homes, this is gonna be very hard to regulate.” At the business level, it was recommended that posting better warning signs of such products to inform consumers of what these products contain and their health risks could positively increase prevention messaging in the community.

Overall, the majority of adult and youth focus group participants and interviewees had not seen any form of prevention efforts in the community and believed that there should be policies and more programs in place to prevent youth from accessing gutka and paan masala. In addition, most focus group participants and interviewees did not know where to gain health information regarding gutka/paan masala use or know of available cessation programs targeted to for these products. A few adult Bhutanese focus group participants and one Burmese interviewee stated that they saw more health warnings on the use of gutka and paan masala back in their home countries on TV, before movie screenings, or posted in the community.

## Discussion

This is the first qualitative study to explore use of gutka and paan masala among Bhutanese and Burmese communities in the southeastern U.S. The resulting data found mixed levels of knowledge and perceptions of harm among focus group participants and interviewees. A qualitative study by Hrwyna and colleagues found similar results in the differing opinions on the use of gutka and paan masala [18]. In this study, focus group participants debated the idea that these products either were beneficial or not at all and these products were mostly used in social gatherings or after meals; additionally, several of study participants perceived some types of paan masala were good (e.g., sweet paan) and others that were bad (e.g., tobacco paan) [18].

The various perceived harms identified by Bhutanese and Burmese participants in our study (e.g., tooth decay or cancer) also mirrored the findings from Hrywna and colleagues’ study. The misclassification of gutka and paan masala may also have played a role in the varying levels of knowledge and perceived harm. The Burmese community translates gutka and paan masala as “koon yar” and “koon baung,” respectively. Bhutanese communities name these products as “supari.” The participants and users of these products may not be able to distinguish the differences between the two and may have confused one for another. It is important to further understand how cultural differences contribute to the continuing use and perception of these products as one group may have preference/acceptance of one product over the other. Additionally, it is important to note the discrepancies between the level of knowledge between the Bhutanese and Burmese communities as there were varying levels of knowledge of the ingredients in both gutka and paan masala. This knowledge would assist in creating targeted community prevention tools and resources.

Both focus group participants and interviewees indicated that gutka and paan masala were widely available and easily accessible in the Clarkston community. Particularly, there were no formal ID checks when individuals purchased these products, therefore creating outlets where youths could access these products more easily and increasing the chances of using these products at an early age. Most of these products could be easily purchased in ethnic stores and grocery markets, likely further exposing more residents to gutka and paan masala. Although most participants indicated that individuals needed to be 18 years and older to purchase gutka, paan masala, or tobacco, there were still many responses that denoted age ranges from 11 years to over 21 years old as the age for purchase. Other participants reported that there was no age requirement for purchase. The discrepancy in responses indicated that not everyone in the community was fully aware of the legal age for tobacco use since gutka is mostly tobacco-based, and, depending on the user, tobacco can also be added to paan masala. This lack of awareness may again be due to a lack of understanding of the concept of legal minimum age for tobacco use. Participants in the study may not have understood concepts of legal ages, perhaps because their home country either did not have them in place or had younger legal ages that differ from the U.S. There is no legal age defined in the country of Bhutan. However, minors under the age of 18 are prohibited from importing tobacco products for personal use [37, 38]. The country of Myanmar does not define the legal age for tobacco use [39].

A literature review on the social contexts of smokeless tobacco use among South Asians identified promising intervention strategies targeting South Asian immigrants in the U.S such as identifying community-based role models that could promote the reduction of tobacco use, media messaging to change cultural norms, counseling and screening for tobacco use, providing quitline services, and tobacco retail policies [40]. Despite adults being the main group of users, our interviews suggested a rise in youth use of gutka and paan masala. To prevent further increase in the use of gutka and paan masala among youth, schools and community centers should consider the addition of an awareness program on the harms and health risks of using gutka and paan masala that educates school children, parents, teachers, and the general community.

For example, an education program for betel nut chewing for school children was conducted in Papua New Guinea in 30-minute sessions. The 150 school children who attended the program showed an increase in knowledge of the product [41]. It should be noted that the motivation to start or quit, however, was low, suggesting more intensive education was needed [41]. Another clustered randomized trial assessed the effectiveness of a school-based smokeless tobacco intervention among 1,327 Karachi school children. This intervention was an 8-week program in one academic year that included a 30-minute PowerPoint presentation, two posters, one pictorial booklet, and video game on the hazards of use of various tobacco chewing products. The intervention had a significant effect on the improvement of knowledge about the health risks of smokeless tobacco products among the school children [42]. These existing studies suggest possible strategies that can be adopted to educate Bhutanese and Burmese communities on use of gutka and paan masala.

Additionally, there is a need for more prevention strategies to reduce tobacco consumption in the Clarkston community. For example, surveillance questions should be added to state-led surveys such as the Georgia Student Health Survey (GSHS) to improve reporting standards among youth populations. Currently, the GSHS only includes conventional drugs (e.g., tobacco, marijuana, prescription drugs). These questions do not capture smokeless tobacco use among South Asians. It would also be more informative if these surveys include ethnic subgroups in their demographics to better understand the prevalence of use in different communities. Formal age restrictions on gutka and paan masala purchase as well as formal tobacco compliance checks to ensure underaged youths cannot easily access these products should be enforced.

The lack of knowledge of prevention resources and policy may also be related to low literacy levels among most Bhutanese and Burmese individuals [22]. Education and awareness programs should be created, including visually-based promotional materials for the community [32]. Due to the literacy gaps noted in Bhutanese and Burmese communities [22], it would be best to recruit a trusted community member or leader to provide this education or otherwise spearhead an awareness program. In order for Bhutanese and Burmese residents to notice the promotional materials and prevention messages, small focus groups can be conducted to evaluate what kind of messages would be effective in the community. If community members are involved in creating these messages, it may have a greater impact and promote a greater sense of community ownership of the issue. Non-profit organizations and the city can collaborate with local clinics and healthcare providers to spread awareness of gutka and paan masala by displaying community-approved prevention messages in offices and having providers speak to patients about the health risks associated with gutka and paan masala.

At the business level, one recommendation would be to post better warning signs in-language and in English. Most participants stated there were no such signs currently present in the community. This signage would also assist law enforcement in regulating these products. Additionally, warning labels on tobacco products have been shown to be effective in providing health information to users and have been associated with health knowledge [43]. Warning labels have been proven to encourage smokers to quit and discourage others to start smoking as well as encourage cessation. The addition of graphic warning labels compared to text only labels have also discouraged youth users to continue smoking or start smoking [44].

It is also important that business owners and merchants are educated regarding the regulations governing important and the sale of tobacco products. Culturally-specific tobacco products must be addressed at the local, state and federal level to advance existing and future tobacco control efforts [40]. Many state and local governments require retailers to attend trainings prior to receiving their licenses [45], however when these businesses are fined or penalized, there is a lack of follow up on how retailers can continue to stay compliant with the law. Even though these culturally specific tobacco products are not covered under current tobacco laws, by definition they are still tobacco products and retailers should be aware of these distinctions.

### Strengths and limitations

Strengths of the study include the large number of focus groups done with both adults and youths from Bhutanese and Burmese communities as well as several key informant interviews with community members. We also acknowledge the main limitations in this study. We did not collect quantitative sociodemographic data of participants in the study. Additionally, since the Burmese community is made up of over 33 different ethnic groups with distinctly different dialects, occasionally the interpreters had difficulties interpreting or translating certain phrases and words. Future studies should include another focus group solely focused on the Burmese community disaggregated by sub-ethnic groups (e.g., Karen, Chin) to understand which group may be using gutka or paan masala more. This would give better insight on the prevalence of gutka/paan masala use among different sub-ethnic groups within the Burmese community. We did not ask questions about personal use of gutka or paan masala during focus groups for adults and youths. Survey data should be collected in future research to assess the prevalence of use, likelihood of use, perception of harm, acculturation, and access.

## Conclusions

Our study represents the first effort to explore use of gutka and paan masala use among Bhutanese and Burmese communities in the southeastern U.S. Our findings indicated that culturally-relevant awareness and education programs as well as health promotion materials regarding gutka and paan masala are much needed in Bhutanese and Burmese communities. More regulatory actions are needed, such as better warning signs in businesses to inform customers of ingredients in these products and their health risks, age restrictions on gutka and paan masala purchase, and compliance checks.

## Supporting information

S1 Appendix Focus group questions. (DOCX)

S2 Appendix Key informant interview questions. (DOCX)
