# Supplementary material for: A qualitative study of gutka and paan masala use among Bhutanese and Burmese migrants in Georgia
Source: PLoS One. 2020 Aug 7;15(8):e0237266. doi: 10.1371/journal.pone.0237266 (PMC7413493; doi:10.1371/journal.pone.0237266)
Supplement: S1 Appendix — (DOCX) [file pone.0237266.s001.docx]

**S1 Appendix: Focus group questions**

***Knowledge and Perceptions of Harm of Gutka and Paan Masala***

1. How would you describe gutka/paan masala? What are some commonly-used brands?
2. Can you tell me about the ingredients in gutka? In paan masala? What type of flavors are there for these products?
3. Can you tell me about the history of these products?
4. Can you tell me about your knowledge of betel nut? Slaked lime? Tobacco?
5. Can you tell me about your thoughts on the health risks and harms of gutka? Of paan masala?
6. What are your concerns, if any, of the consequences of gutka/paan masala use in your community?

***Patterns of and Reasons for Use***

1. How common is gutka use in your community? What about paan masala? Tell me more about it.
2. In your opinion, is the use of gutka/paan masala prevalent among youth and/or adults? Who uses these products the most? Why do people use them? What makes these products attractive?

***Access to Gutka and Paan Masala***

1. Where can you buy or get gutka/paan masala?
2. How is gutka/paan masala sold and promoted? Is it placed behind or on the counter?
3. How easy is it for youth/adults to access gutka/paan masala?
4. How old do you have to be to buy gutka/paan masala? To buy tobacco?
5. Have you ever attempted to buy gutka/paan masala? Please describe your experience.
6. From your experience, has any clerk or cashier ever checked your ID when you buy gutka/paan masala?

***Resources for Cessation and Prevention***

1. What has your experience been with gutka/paan masala in your household? Is it in visible sight for children to see?
2. What kind of conversations have you had with your parents/children about gutka/paan masala?
3. What kind of efforts are available in your community addressing gutka/paan masala use in terms of resources for prevention and cessation?
4. Have you ever seen any type of advertisement, poster, campaign warning about the risks associated with gutka/paan masala? Please describe your experience, if any, with them.
5. In your opinion, what would be the best way to reach out to your community in regard to gutka/paan masala prevention?
